# Cat ownership, cat allergen exposure, and trajectories of sensitization and asthma throughout childhood

**DOI:** 10.1016/j.jaci.2017.09.030

**Published:** 2018-02

**Authors:** Healson Ihuoma, Danielle C. Belgrave, Clare S. Murray, Philip Foden, Angela Simpson, Adnan Custovic

**Affiliations:** a Division of Infection, Immunity and Respiratory Medicine, Faculty of Biology, Medicine and Health, Manchester Academic Health Sciences Centre, University of Manchester and University Hospital of South Manchester NHS Foundation Trust, Manchester, United Kingdom; b Section of Paediatrics, Department of Medicine, Imperial College, London, United Kingdom

To the Editor:

For almost 2 decades, cat exposure has been linked to a number of different and often contradictory outcomes. Several birth cohorts reported increased risk of cat-specific sensitization in preschool children with increasing early-life cat allergen exposure.^1^, ^2^, ^3^ In contrast, a protective effect of high allergen exposure on cat sensitization, with a bell-shaped dose-response relationship, has been reported in cross-sectional studies in older children and adults.^4^, ^5^ Similar inconsistencies have been reported on the association between cat ownership and cat-specific sensitization.^1^, ^6^, ^7^ We hypothesized that the effect of early-life cat exposure on sensitization differs (1) over time, rendering the generalization of effects from cross-sectional analyses at specific age points potentially misleading; (2) for different allergenic proteins from cat; and (3) between children with different risk of allergy.

To address our hypotheses, we investigated the effect of cat exposure in the first year of life on longitudinal trajectories of sensitization and asthma throughout childhood in a population-based birth cohort in which skin prick tests (SPTs) and IgE to cat allergens Fel d 1, 2, and 4 (ImmunoCAP ISAC) were available at 6 time points (ages 1, 3, 5, 8, 11, and 16 years). We defined SPT sensitization as a wheal diameter of greater than or equal to 3 mm; for component-resolved diagnostics (IgE/CRD), we considered a child being sensitized if the IgE level was more than 0.3 ISU to at least 1 component. We ascertained cat ownership in infancy using questionnaires, and quantitated Fel d 1 in dust samples collected in homes within the first year of life using ELISA (μg/g). We carried out longitudinal analyses using generalized estimation equations (GEEs). We generated prototypical trajectories of sensitization from infancy to adolescence on the basis of mean predicted values of the multivariate GEE model, with an interaction between cat ownership and time.

We analyzed data for 1004 of 1051 children in the observational cohort who had confirmed exposure in infancy, and at least 1 valid sensitization measurement. Table E1 in this article's Online Repository at www.jacionline.org presents the demographic and clinical characteristics of study participants. In cross-sectional analyses, cat ownership in infancy was associated with a significantly higher risk of cat sensitization in preschool age, but not thereafter (see Fig E1 in this article's Online Repository at www.jacionline.org). In the multivariable longitudinal model (Fig 1; see Table E2 in this article's Online Repository at www.jacionline.org), early-life cat ownership significantly increased the risk of sensitization to cat (odds ratio [OR], [95% CI]: SPT, 2.50 [1.37-4.55], *P* = .003; IgE/CRD, 3.13 [1.62-6.07], *P* = .001). However, there was a significant interaction between early-life cat ownership and time, in that compared with cat owners, among children without a cat the annual increase in the rate of SPT sensitization from 1 to 16 years was 6% higher (95% CI, 1% to 11%; *P* = .02), and for IgE/CRD sensitization 8% higher (95% CI, 2% to 14%; *P* = .005). Most children with a cat in the home who developed cat sensitization during childhood did so by age 1 year. After age 1 year, the sensitization rate among cat owners either increased very slowly (SPT, Fig 1, *A*), or remained unchanged (IgE/CRD, Fig 1, *B*). In contrast, for children without a cat, the preschool sensitization rate was low, but their trajectory over time was markedly different, with a significantly higher increase in sensitization with increasing age. By adolescence, there was no difference in the point prevalence of cat sensitization between cat owners and those not owning a cat (Fig 1).

**Fig 1:**
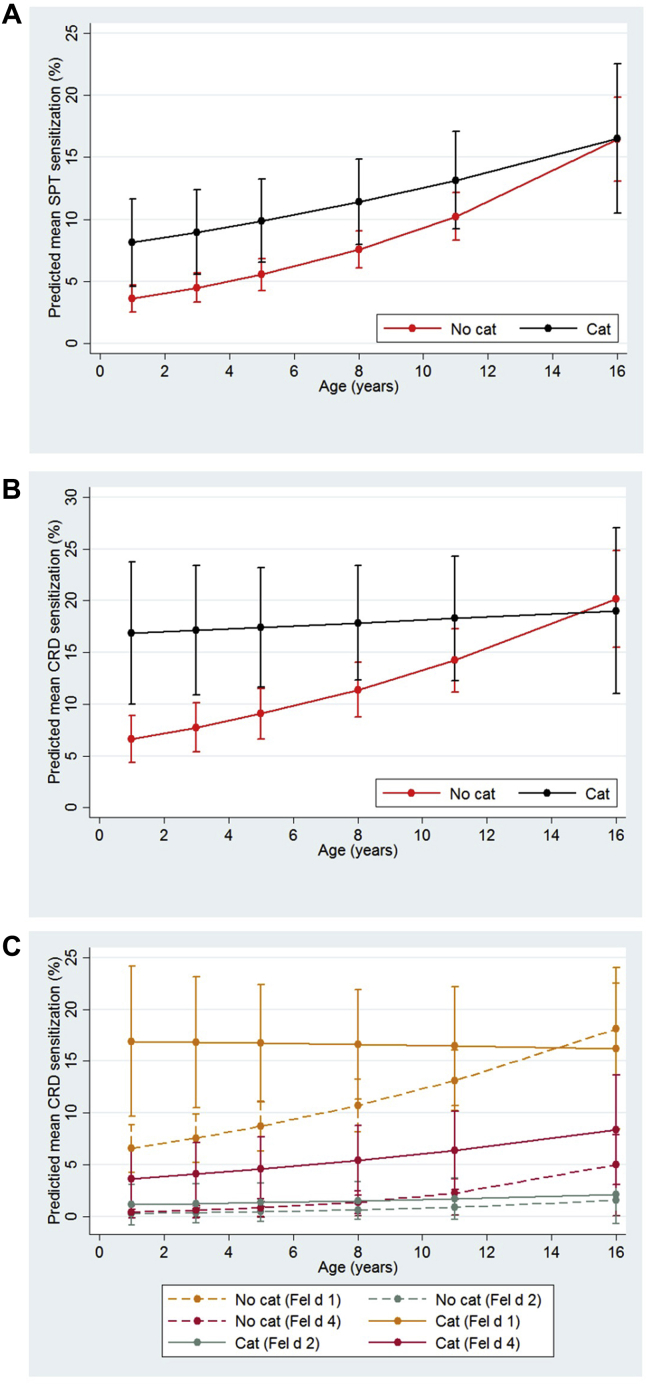
Longitudinal trajectories of cat sensitization among children who lived in a home with a cat in early life and those who did not. Predicted value of mean response is shown in graphical format along with 95% CIs. **A,** SPT sensitization. **B,** CRD sensitization to cat. **C,** CRD components.

Cat ownership significantly increased the risk of sensitization to Fel d 1 and Fel d 4, but not Fel d 2, with a significant interaction between cat ownership and time in relation to the development of IgE to Fel d 1 (*P* = .008), marginal for Fel d 4 (*P* = .06), but not for Fel d 2 (*P* = .39) (Fig 1, *C*).

We measured Fel d 1 in 939 homes. In cross-sectional analyses, an increase in Fel d 1 exposure in infancy significantly increased the likelihood of sensitization in preschool age, but not thereafter (see Table E3 in this article's Online Repository at www.jacionline.org). In the multivariable GEE model, there was a significant association between early-life Fel d 1 exposure and the development of sensitization to cat (see Table E4 in this article's Online Repository at www.jacionline.org); the increase in risk per logarithmic unit increase in Fel d 1 concentration was 15% for SPT (95% CI, 4% to 28%; *P* = .008) and 22% for IgE/CRD (95% CI, 10% to 36%; *P* < .001). However, as with cat ownership, we observed a significant interaction between Fel d 1 exposure and time, in that the effect of early-life exposure significantly decreased with increasing age (SPT: OR [95% CI], 0.99 [0.98-1.00], *P* = .04; IgE/CRD: 0.98 [0.98-0.99], *P* < .001).

Sensitization rates were consistently higher among high-risk children of atopic parents, but the rate of change over time in relation to cat ownership or allergen exposure did not significantly differ between the high- and low-risk children (Fig 2; Table E2, Table E3, Table E4). Finally, there was no effect of cat ownership on sensitization to allergens other than cat, and no significant effect on asthma (see Fig E2 in this article's Online Repository at www.jacionline.org). Fel d 1 exposure in infancy was not significantly associated with the development of asthma (0.98 [0.89-1.07]; *P* = .61). The absence of any association between cat exposure and asthma is consistent with a pooled analysis of 11 European birth cohorts,^6^ and suggests that early-life exposure to cat exerts cat allergen-specific immune responses.

**Fig 2:**
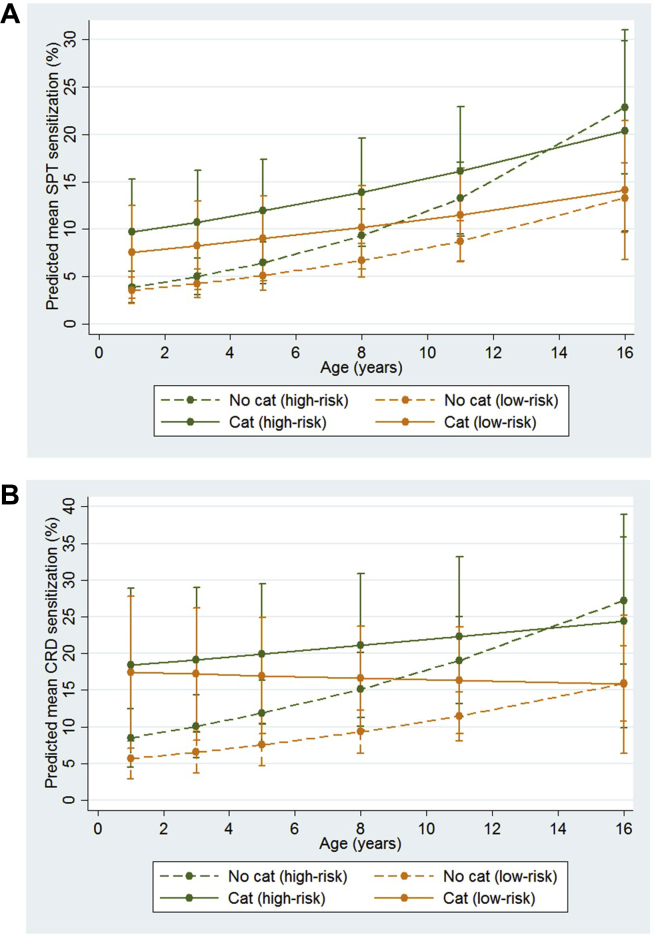
Longitudinal trajectories of cat sensitization among high- and low-risk children who lived in a home with a cat in early life and those who did not. Predicted value of mean response is shown in graphical format along with 95% CIs. High-risk group: Both biological parents with at least 1 positive SPT result at recruitment. **A,** SPT sensitization. **B,** IgE/CRD sensitization.

In our population, 18% of cat owners removed their cat between pregnancy and the first birthday of their child. When we adjusted our analyses for contemporaneous exposure, there was very little difference in the results. Limitations of our study are discussed in this article's Online Repository at www.jacionline.org.

Our findings highlight the changing nature of the association between early-life cat exposure and specific sensitization during childhood, and the key role of the time of the assessment of outcomes. These results can explain most inconsistencies in the previous literature. For example, most reports from birth-cohort studies have assessed sensitization in preschool age, and have identified cat ownership as a risk factor, along with a linear dose-response relationship between cat allergen exposure and specific sensitization.^1^, ^2^, ^3^, ^8^ Cross-sectional and case-control studies in older children and adults have found either no association or a protective effect of cat exposure.^4^, ^5^, ^9^ This is entirely consistent with our sensitization trajectories. The sensitization rate at age 16 years in our study was numerically lower among children who had a cat in early life, but this difference was not statistically significant. We propose that if we extrapolate our data to adulthood, with projected sensitization rates of 25% to 30%, a significant protective effect of cat ownership would be seen. Thus, exposure to cat can confer either an increase in risk, or protection, or will have no effect, depending on the age of the assessment, study design, and the choice of the study population. Therefore, the fact that the findings of previous studies are apparently contradictory does not make them incorrect, but is a consequence of markedly different trajectories of cat sensitization through life-course between early-life cat owners compared with individuals without a cat.

## Figures and Tables

**Fig E1:**
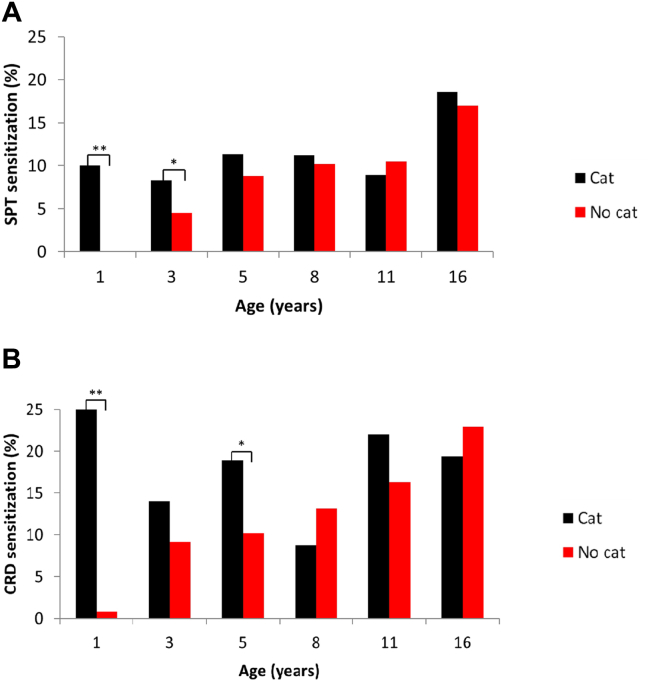
Cross-sectional association between cat ownership status during pregnancy and cat-specific sensitization (SPT, sIgE, CRD) during childhood. **A,** SPT sensitization. **B,** sIgE/CRD sensitization. *P < .05. **P < .001.

**Fig E2:**
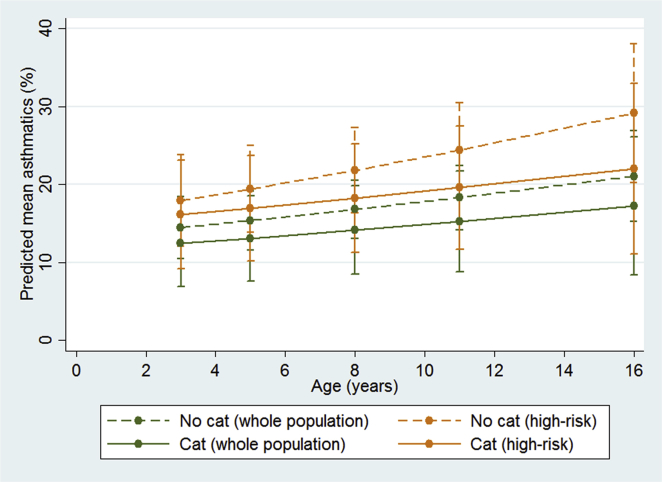
Prototypical longitudinal trajectory of asthma development among children who owned a cat and those who did not own a cat in early life. Predicted value of mean response is shown in graphical format along with 95% CIs.

**Table E1:** Demographic and clinical characteristics of the study population

Characteristic	Age 1 y	Age 3 y	Age 5 y	Age 8 y	Age 11 y	Age 16 y
Sex (male)	482 of 878 (54.9)	489 of 908 (53.9)	508 of 932 (54.5)	483 of 879 (55.0)	429 of 791 (54.2)	346 of 648 (53.4)
Cat owner	196 of 878 (22.3)	197 of 908 (21.7)	209 of 932 (22.4)	207 of 879 (23.6)	209 of 791 (26.4)	175 of 648 (27.0)
High risk	266 of 853 (31.2)	289 of 890 (32.5)	285 of 907 (31.4)	266 of 854 (31.1)	240 of 776 (30.9)	202 of 631 (32.0)
Maternal asthma	111 of 877 (12.7)	118 of 907 (13.0)	125 of 931 (13.4)	111 of 878 (12.6)	99 of 790 (12.5)	80 of 648 (12.3)
Maternal smoking	118 of 878 (13.4)	166 of 907 (18.3)	176 of 929 (19.0)	148 of 878 (16.9)	116 of 790 (14.7)	80 of 646 (12.4)
Allergic sensitization	35 of 318 (11.0)	166 of 821 (20.2)	226 of 814 (27.8)	249 of 798 (31.2)	234 of 694 (33.7)	272 of 492 (55.3)
Asthma	N/A	121 of 766 (15.8)	191 of 787 (24.3)	148 of 743 (19.9)	153 of 675 (22.7)	108 of 521 (20.7)
Current wheeze	233 of 836 (27.9)	194 of 889 (21.8)	195 of 892 (21.9)	152 of 876 (17.4)	138 of 789 (17.5)	101 of 633 (16.0)
Cat SPT sensitized	7 of 318 (2.2)	43 of 816 (5.3)	76 of 812 (9.4)	83 of 798 (10.4)	70 of 694 (10.1)	95 of 545 (17.4)
CRD sensitized	10 of 144 (6.9)	18 of 175 (10.3)	62 of 494 (12.6)	56 of 466 (12.0)	69 of 397 (17.4)	69 of 310 (22.3)
Fel d 1 sensitized	9 of 144 (6.3)	17 of 175 (9.7)	60 of 494 (12.2)	52 of 466 (11.2)	62 of 397 (15.6)	62 of 310 (20.0)
Fel d 2 sensitized	0 of 144	1 of 175 (0.6)	5 of 494 (1.0)	1 of 466 (0.2)	9 of 397 (2.3)	6 of 310 (1.9)
Fel d 4 sensitized	3 of 144 (2.1)	4 of 175 (2.3)	10 of 494 (2.0)	7 of 466 (1.5)	17 of 397 (4.3)	18 of 310 (5.8)

**Table E2:** Association between cat ownership in early life and the development of sensitization to cat: Longitudinal GEE model

Predictors of sensitization	OR	95% CI	*P* value
SPT—whole population			
Sex (male)	1.77	1.22-2.58	.003
Maternal age at birth (y)	1.05	1.01-1.09	.008
Cat ownership in early life	2.50	1.37-4.55	.003
Time (y)	1.12	1.09-1.15	<.001
Interaction—Cat ownership and time	1.06	1.01-1.11	.02
SPT—high-risk			
Sex (male)	1.40	0.81-2.41	.23
Maternal age at birth (y)	1.03	0.98-1.08	.23
Cat ownership in early life	2.86	1.25-6.56	.01
Time (y)	1.14	1.10-1.19	<.001
Interaction—Cat ownership and time	1.08	1.01-1.15	.03
SPT—low-risk			
Sex (male)	2.09	1.25-3.50	.005
Maternal age at birth (y)	1.06	1.01-1.11	.01
Cat ownership in early life	2.37	0.99-5.65	.05
Time (y)	1.10	1.07-1.14	<.001
Interaction—Cat ownership and time	1.05	0.98-1.13	.16
CRD—whole population			
Sex (male)	1.37	0.88-2.13	.16
Maternal age at birth (y)	1.06	1.01-1.10	.009
Cat ownership in early life	3.13	1.62-6.07	.001
Time (y)	1.09	1.06-1.12	<.001
Interaction—Cat ownership and time	1.08	1.02-1.14	.005
CRD—high-risk			
Sex (male)	0.79	0.41-1.52	.48
Maternal age at birth (y)	1.05	0.99-1.12	.10
Cat ownership in early life	2.63	1.06-6.52	.04
Time (y)	1.10	1.06-1.14	<.001
Interaction—Cat ownership and time	1.07	1.00-1.15	.06
CRD—low-risk			
Sex (male)	2.14	1.17-3.92	.01
Maternal age at birth (y)	1.06	1.00-1.12	.04
Cat ownership in early life	3.92	1.48-10.35	.006
Time (y)	1.08	1.04-1.13	<.001
Interaction—Cat ownership and time	1.09	1.01-1.18	.04
Fel d 1			
Sex (male)	1.42	0.91-2.22	.13
Maternal age at birth (y)	1.06	1.01-1.11	.009
Cat ownership in early life	3.20	1.60-6.37	.001
Time (y)	1.08	1.05-1.11	<.001
Interaction—Cat ownership and time	1.08	1.02-1.15	.008
Fel d 2			
Sex (male)	0.83	0.21-3.23	.79
Maternal age at birth (y)	1.06	0.95-1.19	.31
Cat ownership in early life	4.39	0.42-45.69	.22
Time (y)	1.12	1.00-1.25	.04
Interaction—Cat ownership and time	1.08	0.91-1.27	.39
Fel d 4			
Sex (male)	0.92	0.39-2.18	.84
Maternal age at birth (y)	0.98	0.89-1.08	.70
Cat ownership in early life	9.54	1.76-51.78	.009
Time (y)	1.18	1.07-1.30	.001
Interaction—Cat ownership and time	1.11	1.00-1.24	.06

**Table E3:** Cross-sectional analysis: The association between early-life Fel d 1 exposure and sensitization

Age	OR	95% CIs	*P* value
SPT—whole population			
**1 y**	**1.71**	**1.20-2.45**	**.003**
**3 y**	**1.17**	**1.04-1.32**	**.009**
5 y	1.04	0.94-1.15	.50
8 y	1.01	0.91-1.12	.90
11 y	0.98	0.88-1.10	.79
16 y	1.03	0.93-1.13	.59
SPT—high-risk			
**1 y**	**1.71**	**1.20-2.45**	**.003**
3 y	1.11	0.92-1.33	.28
5 y	1.01	0.86-1.18	.90
8 y	0.93	0.79-1.10	.42
11 y	0.89	0.72-1.10	.29
16 y	0.95	0.81-1.11	.50
SPT—low-risk			
1 y	—	—	—
**3 y**	**1.22**	**1.05-1.43**	**.01**
5 y	1.06	0.93-1.21	.41
8 y	1.07	0.93-1.21	.35
11 y	1.03	0.90-1.19	.63
16 y	1.09	0.96-1.23	.19
CRD—whole population			
**1 y**	**1.32**	**1.03-1.70**	**.03**
3 y	1.07	0.87-1.32	.53
**5 y**	**1.15**	**1.03-1.28**	**.01**
8 y	0.99	0.88-1.12	.93
11 y	1.05	0.93-1.18	.45
16 y	1.00	0.89-1.12	.98
CRD—high-risk			
**1 y**	**1.32**	**1.03-1.70**	**.03**
3 y	1.08	0.88-1.34	.46
**5 y**	**1.20**	**1.00-1.44**	**.05**
8 y	0.89	0.71-1.10	.28
11 y	0.97	0.81-1.17	.74
16 y	0.85	0.70-1.04	.12
CRD—low-risk			
1 y	—	—	—
3 y	0.79	0.13-4.59	.79
5 y	1.13	0.98-1.31	.08
8 y	1.05	0.91-1.22	.51
11 y	1.12	0.95-1.31	.17
16 y	1.11	0.96-1.28	.17
Fel d 1—whole population			
**1 y**	**1.29**	**0.99-1.67**	**.06**
3 y	1.08	0.87-1.34	.48
**5 y**	**1.12**	**1.00-1.25**	**.05**
8 y	0.98	0.87-1.12	.81
11 y	1.05	0.93-1.18	.44
16 y	0.99	0.88-1.12	.87
Fel d 1—high-risk			
**1 y**	**1.29**	**0.99-1.67**	**.06**
3 y	1.10	0.88-1.36	.41
5 y	1.16	0.96-1.39	.12
8 y	0.92	0.74-1.15	.47
11 y	0.96	0.79-1.16	.67
16 y	0.85	0.70-1.05	.13
Fel d 1—low-risk			
1 y	—	—	—
3 y	0.79	0.13-4.59	.79
5 y	1.11	0.96-1.28	.17
8 y	1.02	0.87-1.19	.85
11 y	1.13	0.96-1.33	.13
16 y	1.10	0.95-1.29	.21

Values in boldface are significant results.

**Table E4:** The association between early-life Fel d 1 exposure and the longitudinal trajectory of sensitization to cat (longitudinal GEE model)

Predictors of sensitization	OR	95% CI	*P* value
SPT—whole population			
Sex (male)	1.73	1.17-2.56	.006
Maternal age at birth	1.05	1.01-1.09	.01
Fel d 1 exposure	1.15	1.04-1.28	.008
Time (age)	1.11	1.08-1.13	<.001
Interaction—Fel d 1 exposure and time	0.99	0.98-1.00	.04
SPT—high-risk			
Sex (male)	1.46	0.82-2.58	.20
Maternal age at birth	1.04	0.99-1.10	.14
Fel d 1 exposure	1.19	1.02-1.38	.03
Time (age)	1.13	1.10-1.17	<.001
Interaction—Fel d 1 exposure and time	0.98	0.97-1.00	.02
SPT—low-risk			
Sex (male)	1.97	1.15-3.37	.01
Maternal age at birth	1.06	1.00-1.11	.04
Fel d 1 exposure	1.14	0.99-1.31	.08
Time (age)	1.09	1.05-1.12	<.001
Interaction—Fel d 1 exposure and time	1.00	0.99-1.00	.39
CRD—whole population			
Sex (male)	1.28	0.81-2.02	.30
Maternal age at birth	1.06	1.02-1.11	.006
Fel d 1 exposure	1.22	1.10-1.36	<.001
Time (age)	1.08	1.05-1.11	<.001
Interaction—Fel d 1 exposure and time	0.98	0.98-0.99	<.001
CRD—high-risk			
Sex (male)	0.79	0.40-1.57	.50
Maternal age at birth	1.07	1.00-1.14	.06
Fel d 1 exposure	1.22	1.04-1.43	.02
Time (age)	1.09	1.05-1.13	<.001
Interaction—Fel d 1 exposure and time	0.98	0.97-0.99	.003
CRD—low-risk			
Sex (male)	1.93	1.04-3.61	.04
Maternal age at birth	1.06	1.00-1.13	.04
Fel d 1 exposure	1.22	1.06-1.41	.007
Time (age)	1.06	1.02-1.10	.003
Interaction—Fel d 1 exposure and time	0.99	0.98-1.00	.05
Fel d 1 IgE—whole population			
Sex (male)	1.33	0.83-2.12	.23
Maternal age at birth	1.07	1.02-1.12	.005
Fel d 1 exposure	1.21	1.09-1.35	<.001
Time (age)	1.07	1.04-1.10	<.001
Interaction—Fel d 1 exposure and time	0.98	0.98-0.99	.001
Fel d 1 IgE—high-risk			
Sex (male)	0.77	0.39-1.53	.46
Maternal age at birth	1.08	1.00-1.15	.04
Fel d 1 exposure	1.24	1.05-1.46	.01
Time (age)	1.09	1.04-1.13	<.001
Interaction—Fel d 1 exposure and time	0.98	0.97-0.99	.005
Fel d 1 IgE—low-risk			
Sex (male)	2.19	1.14-4.20	.02
Maternal age at birth	1.06	1.00-1.13	.05
Fel d 1 exposure	1.19	1.03-1.38	.02
Time (age)	1.04	1.01-1.08	.02
Interaction—Fel d 1 exposure and time	0.99	0.98-1.00	.10

## Discussion

### Limitations

There were some differences between children included and excluded from our analysis (eg, at age 11 years, children who were excluded were more likely to be sensitized). However, none of these differences were consistent across different ages and outcomes. Although this is unlikely to have influenced the results, we cannot unequivocally exclude this possibility.

We reviewed only a subgroup of children at age 1 year for practical reasons. We focused our follow-up on infants with both atopic parents, and those whose 2 parents were nonatopic. The data were not missing at random, and we cannot exclude the possibility that this may have introduced bias in our analyses. However, exclusion of age 1 year data from the longitudinal analysis did not make any material difference to sensitization trajectories (data not shown, available on request). We believe that presenting the age 1 year data is important because these results suggest that among high-risk children who live in a home with a cat, most of those who develop sensitization to cat do so by age 1 year.

The nature of the data collection makes it difficult to differentiate between the effect of exposure to allergen to that of exposure to animal. The finding that significant effects of cat ownership and cat allergen exposure were almost identical and confined to cat allergen-specific sensitization (but not sensitization to other allergens or asthma) suggests that the observed effects are related to allergen exposure, rather than other factors potentially related to cat ownership (eg, exposure to endotoxin). The absence of any association between cat ownership and asthma suggests that early-life exposure to cat exerts cat allergen-specific immune responses.

When assessing allergen exposure, the relative importance of the choice of sampling site is unknown, and there has been little uniformity between studies. There is a possibility that living room floor Fel d 1 concentration may not be the best index of allergen exposure. However, because the correlation between allergen levels measured in different reservoirs is high, it is unlikely that the use of other indices of exposure would materially alter our findings.

We could not differentiate between the effect of exposure during pregnancy from that in early life. However, most families who keep cats in home during pregnancy remain cat owners after the birth of their child. In our population, 18% of cat owners removed their cat between pregnancy and the first birthday of their child. When we adjusted our analyses for contemporaneous exposure, there was very little difference in the results (data not shown, available on request).
